# Frequency and Density-Dependent Selection on Life-History Strategies – A Field Experiment

**DOI:** 10.1371/journal.pone.0001687

**Published:** 2008-02-27

**Authors:** Tapio Mappes, Minna Koivula, Esa Koskela, Tuula A. Oksanen, Tiina Savolainen, Barry Sinervo

**Affiliations:** 1 Department of Biological and Environmental Science, University of Jyväskylä, Jyväskylä, Finland; 2 MTT Biotechnology and Food Research, Jokioinen, Finland; 3 Department of Ecology and Evolutionary Biology, University of California Santa Cruz, Santa Cruz, California, United States of America; University of Exeter, United Kingdom

## Abstract

Negative frequency-dependence, which favors rare genotypes, promotes the maintenance of genetic variability and is of interest as a potential explanation for genetic differentiation. Density-dependent selection may also promote cyclic changes in frequencies of genotypes. Here we show evidence for both density-dependent and negative frequency-dependent selection on opposite life-history tactics (low or high reproductive effort, RE) in the bank vole (*Myodes glareolus*). Density-dependent selection was evident among the females with low RE, which were especially favored in low densities. Instead, both negative frequency-dependent and density-dependent selection were shown in females with high RE, which were most successful when they were rare in high densities. Furthermore, selection at the individual level affected the frequencies of tactics at the population level, so that the frequency of the rare high RE tactic increased significantly at high densities. We hypothesize that these two selection mechanisms (density- and negative frequency-dependent selection) may promote genetic variability in cyclic mammal populations. Nevertheless, it remains to be determined whether the origin of genetic variance in life-history traits is causally related to density variation (e.g. population cycles).

## Introduction

A fundamental problem in evolutionary biology is to find mechanisms maintaining additive genetic variation in natural populations [1], [2]. According to evolutionary theory, selection should reduce genetic variation especially in the traits that are closely associated with fitness [1]. However, many species still exhibit large genetic variation in fitness-related life-history traits [3]–[6]. At least five selection mechanisms are hypothesized to maintain genetic variation in nature: mutation-selection balance, heterosis, antagonistic pleiotropy, negative frequency-dependent selection (advantage of rare genotype) and environmental heterogeneity [2], [7]. Many theoretical analyses have focused on the latter two selection mechanisms (e.g. recently [8]), and these models predicts that, for example, negative frequency-dependent selection can be more common in natural populations as previously recognized. Still, all empirical studies exclusively consist of polymorphic populations, where genetic colour morphs are evident (e.g in plants, fish and reptiles) [9]–[11]. In polymorphic systems it has been more obvious to test whether the fitness of individuals depends on their neighbours' genotype. Among the organisms without visible polymorphism, but which still have large genetic variation in important fitness traits (e.g. in life-history traits), negative frequency-dependent selection (advantage of rare genotype) has not yet received wide attention.

Here we aimed to test empirically two selection mechanisms: negative frequency-dependent selection and environmental heterogeneity (density-dependent selection), which are predicted to maintain genetic variation in natural populations [12]–18. Selection was studied in the bank vole (*Myodes glareolus*), a small mammal with high phenotypic and genetic variation in life-history traits, such as reproductive effort, offspring size and number [19]. Significant temporal variation for density-dependent selection is promoted by large seasonal [20] and 3–4 year cyclic density variation in this species [21]. Furthermore, competition between territorial bank vole females is a major mechanism determining their breeding success which, especially at high densities, leads to large variation in relative fitness of individuals [22]–[24]. Together these selective environments could facilitate the origin and existence of opposite life-history tactics whose success would depend both on the current environment and the frequency of opposite tactic in the population. This idea is supported by studies with side-blotched lizards (*Uta stansburiana*) and common lizards (*Lacerta vivipara*), where negative frequency-dependent selection and density variation have been shown to contribute to genetic cycles driven by alternative life-history strategies [25]–[27].

Here we focused on one central life-history trait, reproductive effort (RE) of females, and experimentally studied whether opposite tactics (high vs. low RE) would be favored by different selection pressures. Both the frequencies (rare vs. common) and densities (low vs. high) of the RE tactics were manipulated in large enclosed populations, where their relative survival and breeding success were monitored over the breeding season. Our aim was to experimentally test whether conditions occurring in cyclic small mammal populations could facilitate the origin of opposite life-history tactics which would then promote large genetic variation observed in several traits.

## Results

When studying juvenile recruitment to the adult population, we found that both the number of offspring weaned and proportion of surviving offspring were related to the level of reproductive effort and the frequency and density of alternative RE tactics in adult females (Three-way interactions: *F_1,64_* = 7.16, *P* = 0.009; *F_1,64_* = 7.82, *P* = 0.007, respectively). This indicates that the success of RE tactics differed according to the frequencies and densities, and so in further analyses RE tactics were analyzed separately (Table 1 and 2).

**Table 1:** The effects of frequency and density on number of offspring weaned, and proportion of offspring surviving until weaning in different tactics of reproductive effort.

Low reproductive effort									
	Number of offspring weaned					Proportion of offspring surviving			
		*df_1_*	*df_2_*	*F*	*P*	*df_1_*	*df_2_*	*F*	*P*
Individual level	Frequency	1	18.2	0.19	0.669	1	34.0	0.01	0.935
	Density	1	18.2	7.24	0.015	1	34.0	11.04	0.002
	Freq * Den	1	18.2	2.58	0.126	1	34.0	2.13	0.154
Population level	Frequency	1	9	0.23	0.642	1	9	0.01	0.919
	Density	1	9	8.89	0.015	1	9	17.72	0.002
	Freq * Den	1	9	3.17	0.109	1	9	3.42	0.098

High reproductive effort									
	Number of offspring weaned					Proportion of offspring surviving			
		*df_1_*	*df_2_*	*F*	*P*	*df_1_*	*df_2_*	*F*	*P*
Individual level	Frequency	1	30	2.19	0.149	1	30	4.35	0.046
	Density	1	30	0.08	0.783	1	30	0.01	0.930
	Freq * Den	1	30	4.53	0.042	1	30	6.79	0.014
Population level	Frequency	1	9	2.33	0.161	1	9	3.44	0.097
	Density	1	9	0.82	0.781	1	9	0.01	0.939
	Freq * Den	1	9	4.82	0.056	1	9	5.38	0.046

*Notes:* The analyses of generalized linear mixed models are performed both at the individual level (individual values formed the dependent variables) and at the population level (population means formed the dependent variables). At the individual level, the random effect of population (enclosure) is included in the models (Estimate<0.006, *P*>0.989 in all cases). *df_1_* = numerator df, *df_2_* = denominator df

**Table 2:** Survival differences between the high and low RE females and whether the tactic was rare or common in the population.

Percentage (N) of females surviving to the end of breeding season			
Tactic	Rare	Common	All
Low RE	42.9 (7)	63.0 (27)	58.8 (34)
High RE	66.7 (9)	19.0 (21)	33.3 (30)

Individual level				
	*df_1_*	*df_2_*	*F*	*P*
RE tactic	1	57	0.89	0.348
Frequency	1	57	0.75	0.391
Density	1	26.7	0.57	0.458
RE tactic * Frequency	1	23.6	6.02	0.022
RE tactic *Density	1	57	0.29	0.595
Frequency * Density	1	57	1.64	0.206

Population level				
	*df_1_*	*df_2_*	*F*	*P*
RE tactic	1	15	0.40	0.536
Frequency	1	15	0.40	0.536
Density	1	15	0.24	0.631
RE tactic * Frequency	1	15	4.79	0.045
RE tactic *Density	1	15	0.09	0.772
Frequency * Density	1	15	0.94	0.347

*Notes:* The analyses of generalized linear mixed models are performed both at the individual level (individual values formed the dependent variable) and population level (population means formed the dependent variable). At the individual level, the random effect of population (enclosure) is included in the models (Estimate = 0.046, *P* = 0.339). *df_1_* = numerator df, *df_2_* = denominator df

**Figure 1:**
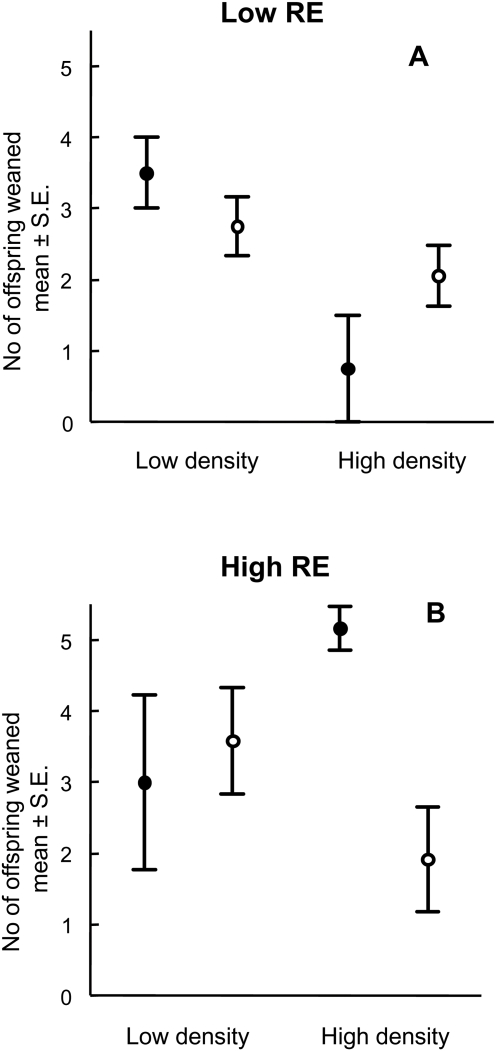
Number of offspring weaned of low (a) and high RE females (b) in different densities and frequencies. Closed circles: rare tactic; Open circles: common tactic in the populations.

**Figure 2:**
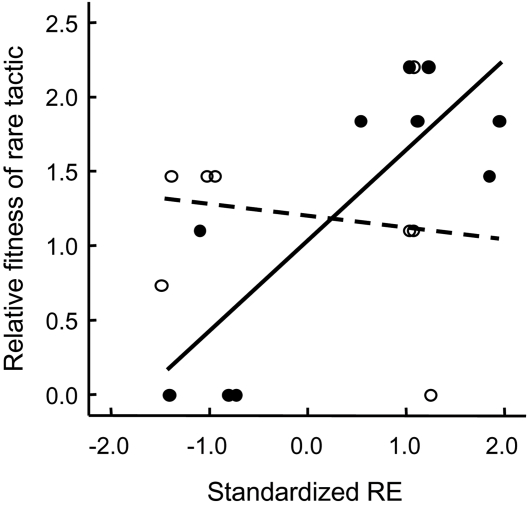
Density-dependent selection for reproductive effort among rare tactics. In the analyses of selection gradients, the relative fitness (number of offspring weaned/mean number of offspring weaned in the population) was estimated in relation to standardized reproductive effort ((RE_i_–RE_mean_)/RE_SD_). Selection gradients indicate directional selection towards higher reproductive effort in high densities (closed circles and solid line: β±SE = 0.60±0.15, *P* = 0.004) but not in low densities (open circles and dashed line: β±SE = −0.08±0.21, *P* = 0.726) (Standardized RE * Density interaction: *F*_1,14_ = 7.34, *P* = 0.017). The selection gradient among common tactics was non-significant (β±SE = 0.33±0.44, *P* = 0.453) and density-independent (Standardized RE * Density interaction: *F*_1,50_ = 0.02, *P* = 0.904).

In particular, females with low RE were favored at low density but RE frequency had no significant effect on their breeding success (Fig. 1A, S1, Table 1, ). Females with high RE were most successful when they were rare in the population and at high density (Fig. 1B, S1, Table 1). Analyses of selection gradients support these results as they indicate directional density-dependent selection towards higher reproductive effort only among rare tactics (Fig. 2). So, negative frequency-dependent selection on higher reproductive effort works effectively in high density populations. Together, these findings suggest that low RE females are successful only in low population densities.

According to life-history theory, the success of alternative RE tactics can also be shaped by the trade-off between RE and reproductive costs. Indeed, significant frequency-dependent survival costs were associated with high RE tactics, especially when they were common in the population (Table 2). Of note, the main results of the density- and frequency-dependent effects on breeding success (Fig. 1A,B, S1, Table 1) were not biased by survival costs, as the survival of females was not yet affected by RE tactic or manipulations of frequencies and densities during their first breeding (Linear logit model, *G*<0.090, *P*>0.663 for all main effects and interactions). Such survival costs were manifested in later breeding episodes.

**Figure 3:**
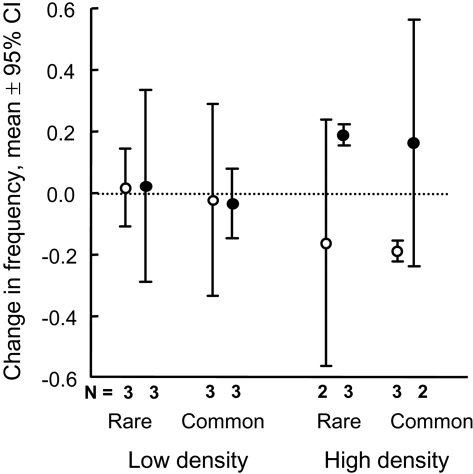
Change in frequencies of low and high RE tactics in the different treatment populations during the breeding season. Frequency of high RE tactics increased significantly when densities were high and initial frequency was low (rare tactic), which simultaneously decreased the frequency of low RE (common tactic). Open circles: low RE; Closed circle: high RE. N = number of replicates (populations).

The survival of mothers and their offspring were monitored until the end of the breeding season to estimate the changes in frequencies of RE tactics at the population level. Frequency of high RE tactics increased significantly when densities were high and initial frequency was low (rare tactic) (*t* = 23.7, *df* = 2, *P* = 0.002), and simultaneously the frequency of low RE females (common and high density) decreased significantly (Fig. 3).

## Discussion

Our results indicate clear negative frequency-dependent and density-dependent selection on different breeding tactics in bank voles. Females with low reproductive effort were especially favored in low densities. Moreover, females with high reproductive effort were most successful when they were rare in high density populations. The different successes of RE tactics were confirmed by both the breeding success and survival of mothers. When studying how selection at the individual level affected the frequencies of tactics at the population level, we found that the frequency of the rare high effort tactic increased significantly in high densities (Fig. 3).

According to these results, we hypothesize that after the crash of a vole population, low RE females have the highest breeding success until the population size increases, at which time high RE females obtain a rare tactic advantage. Another crucial phase of the life history occurs during and after the winter crash phase, which might be beneficial for individuals with low RE. This idea predicts that the frequencies of opposite breeding tactics change according to the seasonal or multi-annual density variations in vole populations.

Negative frequency-dependent selection evidently shows competition between tactics [28]; here it is intraspecific competition between the breeding tactics. In bank voles, high RE females seems to be dominant over the low RE females. As the competition for space is the most important factor affecting breeding success of territorial female bank voles [22], [29]–[31], the high RE females might have a greater ability to occupy and defend their territories especially against low RE females in high densities. In small mammals, territoriality functions to defend food resources and/or offspring against infanticide [32], [33]. The task of future studies is to determine the importance of these two functions for the RE tactics in different densities.

In the current study, females with high reproductive effort have lower survival as predicted by the theory of reproductive costs [34], [35]. These results are consistent with those of our earlier experimental studies, which report a trade-off between reproductive effort (litter size) and mother survival [20], [36]. Interestingly, the reproductive costs of a mother can be affected by other mothers as well as their breeding tactic in the populations. High survival costs of high RE females are evident especially when they compete against the mothers with the same (common) tactic (Table 2). This could be caused by larger food requirements of high RE females. Under intense competition they might not be able to allocate both to reproduction and their own survival.

The density fluctuation causing fitness differences in RE tactics can be annual or multi-annual. It has been hypothesized that even the population dynamics (e.g. population cycles) in small mammals are determined by the fluctuations in genotypes [37]. This hypothesis of intrinsic regulation has not received clear evidence, and so the dynamics of cyclicity (e.g. population crashes) are more evidently affected by external factors e.g. predators and/or food [38]–[40] Still, the “soft” hypothesis of Chitty, that genetic variation is a consequence and not a cause of population cycles, could be valid. However, even after extensive research, this idea is supported only by a few genetic studies in cyclic mammals [41], [42]

In conclusion, we would like to emphasize that it remains to be determined whether the origin of genetic variance in life-history traits is causally related to density variation (e.g. population cycles). Nevertheless, our study demonstrates that selection on alternative life-history strategies would arise from the action of density and frequency cycles.

## Materials and Methods

### Study species

The study species, the bank vole (*Myodes glareolus*), is a small rodent species common in northern Europe [43]. The main habitats are forests and fields, and the diet consists of forbs, shoots, seeds, berries and fungi [44]. The density of breeding females is limited through their territoriality [29], and in high densities the maturation of young females is suppressed by social interaction among females [45]. The patterns and amplitude of density variation show considerable geographical variability, and both stable and cyclic populations are found [46]. In our study area, females give birth to a maximum of four litters during the breeding season, which lasts from late April to September. In addition to large phenotypic [20] and genetic variation in litter size (2–10) and offspring size (1.3–2.5 g), a negative genetic correlation also exists between these traits [19]. Pregnancy lasts for 19–20 days and pups are weaned until the age of three weeks [47]. Bank voles have good trappability, and they are not sensitive to disturbance, which allows monitoring populations by live-trapping.

### Study procedures

Experimental animals originated from artificial selection lines at the University of Jyväskylä. Founder animals for the selection lines were caught in central Finland (62°37′N, 26°20′E). In the truncation selection experiment, two lines were selected according to litter size of females (see details Schroderus et al. 2007, submitted manuscript). The selection experiment was based on the between family selection procedure [48], which prevents the possibility of inbreeding.

Reproductive effort of females is determined here by the formula of [49]: RE = (litter size * mean offspring mass ^0.75^)/mothers post-partum mass ^0.75^). In this formula energy requirements to produce offspring are calculated relative to the allometric requirement of the mother (assuming standard metabolism increases to the 0.75 power of mass for mammals [50]).

The study started by mating a fraction of the females (*n* = 91) obtained from the selection experiment with randomly chosen males of the same line of female (low RE: mean±SE = 0.67±0.02, *n* = 49; High RE: mean±SE = 0.84±0.02, *n* = 42). We further increased the difference between the two groups by choosing only the females with lowest RE (*n* = 39) and highest RE (*n* = 33) for the present experiment. All females were at the same age and in similar reproductive state. Characteristic of these mothers and their offspring and statistical tests are presented in the Table 3. These 72 females gave birth in the laboratory in early July, and after that the females with their new-born individually marked pups were released to 13 large outdoor enclosures (each 0.2 ha) [47]. None of the mothers abandoned their pups during the releasing process. Four males were introduced to each enclosure to keep females in reproductive condition. These males might mate with the females, but we were not able to measure the success of the possible subsequent breeding events.

**Table 3:** Characteristics (mean±SE) of two female tactics (low or high reproductive effort, RE).

			Source of variance (*F*_1,64_)		
	Low RE (*n* = 39)	High RE (*n* = 33)	RE tactic	Frequency	Density
Reproductive effort	0.61±0.02	0.88±0.02	59.56 ***	0.11 ^ns^	1.91 ^ns^
Litter size	3.82±0.14	6.15±0.18	76.36 ***	0.10 ^ns^	3.52 ^ns^
Mean body mass of offspring (g)	1.97±0.03	1.84±0.02	10.60 **	0.02 ^ns^	2.07 ^ns^
Mean head width of offspring (mm)	8.28±0.05	8.09±0.04	6.81 *	0.00 ^ns^	0.07 ^ns^
Post-partum body mass of mother (g)	22.4±0.4	24.6±0.4	6.87 *	0.01 ^ns^	0.27 ^ns^
Post-partum head width of mother (g)	13.8±0.07	13.8±0.06	0.07 ^ns^	1.34 ^ns^	0.10 ^ns^

Random assignment to the different manipulation groups (frequency and density) is tested by three-way ANOVA. All possible two and three-way interactions were non-significant (*P*>0.11).

*** *P*< = 0.001,

** *P*<0.01,

* *P*<0.05

In the enclosures, both the densities (4 or 8 females/0.2 ha) and frequencies (1∶3 (4 enclosures); 3∶1 (4), 2∶6 (2), 6∶2 (3)) of different reproductive tactics (low or high RE) were manipulated (see the design in Table 4). The choice of 4 and 8 females per enclosure to low and high experimental densities respectively, was based on our earlier studies showing that 4–6 females can gain a territory and breed simultaneously in the same enclosure [51].

**Table 4:** The design of the experiment.

	Low density	Low density total	High density	High density total	Overall total
Rare Low RE + Common High RE	1+3 (4)	4+12	2+6 ( 2)	4+12	
Rare High RE + Common Low RE	1+3 (4)	4+12	2+6 (3)	6+18	
Total (Low Re+High RE)		16+16 = 32		22+18 = 40	32+40 = 72

Number of females per enclosure, number of enclosures (in parenthesis) and total number of individuals in each treatment.

The breeding success of all 72 females was studied during one breeding in July. Breeding success was determined by monitoring the number and the proportion of offspring surviving to the weaning age (about 25 days old). For monitoring individual voles, 20 multi-capture live traps were distributed in each enclosure in a 4×5 array with 10 m between the trap stations. Mothers and offspring were trapped at weaning and at the end of the experiment (offspring about three months old) (see details of trapping procedure [24], [52]).

Survival of only 64 females was determined from the beginning of the experiment (early July) to the end of the experiment (early October), as all the individuals in two enclosures escaped before the end of the experiment. The females escaped after the first litter was weaned, so we were able to include all females to the analyses of the breeding success (Table 1 and Fig. 1A,B). Only the successful enclosures (see number of replicates in Fig. 3) were included into the analyses of frequency changes in the population level (see below). No other indications of unsuccessful replicates were found during the experiment.

Selection for reproductive effort was studied using the analyses of selection gradients [48], [53]. Selection gradients (β) were estimated from the linear regression coefficients of relative fitness on the standardized trait. Here the relative fitness (number of offspring weaned/mean number of offspring weaned in the population) was estimated in relation to standardized reproductive effort ((RE_i_–RE_mean_)/RE_SD_).

The survival of mothers (Table 2) and their offspring was monitored until the end of the breeding season to estimate the changes in frequencies of RE tactics at the population level (Fig. 3). Change in frequency was determined by the frequency of the certain breeding tactic (survived mothers and their offspring) at the end of breeding season minus the initial frequency of the breeding tactic in the enclosure.

### Statistical analyses

The data were analysed using SPSS 14.0 for Windows and SAS version 9.1 software. In the generalized linear mixed model analysis (GLMM), the number of offspring weaned, proportion of offspring surviving or mother survival were explained by RE tactic, frequency, density and their interactions. The analyses of GLMM were performed both at the individual level (individual values formed the dependent variables) and population level (population means formed the dependent variables). At the individual level, GLMM allows for the population (enclosure) to be included as a random effect in the models [54].

## Supporting Information

Figure S1 (5.22 MB TIF)
